# Mechanisms of Action and Preliminary Impact of a Stigma-Mitigation Training to Enable Healthcare Discussions About Anal Pleasure and Health in China: A Sequential Explanatory Mixed Methods Pilot Study

**DOI:** 10.1007/s10461-025-04878-6

**Published:** 2025-09-18

**Authors:** Bryan A. Kutner, Kathrine Meyers, Yumeng Wu, Benjamin Lane, Gang Yang, Baichun Hou, Iván C. Balán

**Affiliations:** 1 Albert Einstein College of Medicine & Montefiore Medical Center, Psychiatry Research Institute at Montefiore Einstein, 1225 Morris Park Ave., 4A, Bronx, NY 10461 USA; 2 Aaron Diamond AIDS Research Center, Columbia University Irving Medical Center, New York, NY 10032 USA; 3 China AIDS Walk Special Fund, Inc, Flushing, NY 11355 USA; 4 Center for Translational Behavioral Science, Florida State University College of Medicine, Tallahassee, FL 32310 USA

**Keywords:** Social stigma, Behavior and behavior mechanisms, Intervention study, Implementation science, Theoretical domains framework

## Abstract

Stigma toward anal sex impedes engagement in health services that are vital to Ending the HIV Epidemic. We evaluated a stigma-reduction training to build HIV worker capacity to address anal pleasure and health during HIV service encounters in China. In a sequential explanatory mixed-methods design, we assessed the training’s preliminary impact on anal health promotion activities and, based on the *Theoretical Domains Framework*, mechanisms of action among 51 health workers. We conducted descriptive statistics, bivariate comparisons, and thematic analyses based on pre/post-training online surveys and post-training in-depth interviews. The frequency of anal health promotion activities among the 38 participants who completed pre/post-training surveys increased by 7–8% of the scale range, but not significantly (*p*-values between 0.06 and 0.08). Mechanisms for discussing anal sex (*knowledge*, *skills*, *role responsibility*, *self-efficacy*, *organizational acceptability*, and *positive emotions*) shifted from baseline (5%-36% range), primarily due to boosts in *knowledge* and *skills* (28% and 36%, all *p* < .05). Additional mechanisms of *comfort addressing sexual orientation*, *anal health*, and *sex-related concerns* also increased (4–14% range, all *p* < .05). Qualitative analysis revealed mechanistic themes of heightened empathy, weakened stereotypes, and a shift toward accepting role responsibility to discuss anal sex. Gay and bisexual workers reported notable personal growth to optimize their own anal health. Although knowledge and communication skills increased provider confidence, some participants remained hesitant to initiate discussion during HIV service encounters. For most participants the theoretically-informed training activities operated as hypothesized, to enhance mechanisms that could increase provider-initiated discussion of anal pleasure and health.

## Introduction

Stigma toward anal sex remains a persistent barrier to healthcare access for gay and bisexual men (GBM)^1^ [1–6]. This stigma arises from a broad social process, rooted in power imbalances, whereby the anal sex practices of GBM are labeled as deviant, stereotyped as inherently risky, and met with social separation and distancing. This results in status loss, experiences of discrimination – like neglect of their sexual health within healthcare settings – as well as internalization of stigma and anticipation of future mistreatment [7, 8]. Anal sex stigma manifests through internalized shame, embarrassment and concealment of sexual behavior, and is compounded in some settings by criminalization and social rejection. Across settings, GBM confront systemic gaps in health knowledge related to anal sex, as do the healthcare providers tasked with addressing their sexual health needs. These dynamics create a cyclical burden: GBM internalize stigma and avoid discussions of anal sex with partners and providers, while healthcare workers – often lacking training about anal sex and health [5, 19, 21, 22] – hesitate to broach sexual topics involving anal sex [9–11]. The result is diminished access to health services.

In Chinese society, anal sex is particularly stigmatized due to deeply-rooted traditional values emphasizing family lineage and heterosexual marriage, which are reinforced by Confucian ideals of filial piety and the expectation to procreate [12–14]. Within healthcare settings in China, stigma persists among both providers and clients, with studies showing that most GBM do not disclose their sexual orientation to medical professionals [15, 16], and many healthcare workers lack adequate training or comfort to address the needs of this population [16, 17]. The impact of anal sex stigma on HIV prevention is particularly salient [4, 5, 12, 13], as anal sex, without the protections afforded by access to HIV services [14], is the most proximal behavioral factor for acquiring HIV among GBM globally and in China [15]. However, disclosing sexual behavior is typically a prerequisite for access to HIV prevention services [4, 5]. This complicates equitable distribution of certain biomedical interventions like pre-exposure prophylaxis (PrEP), which has historically required client disclosure of “high risk” anal sex (i.e., condomless intercourse) prior to receiving a prescription [16, 17].

Without a signal from healthcare workers that destigmatized discussion about anal sex is appropriate and welcome, GBM are likely to remain reluctant to disclose the very behavior that could otherwise facilitate their access to HIV prevention methods. However, HIV healthcare workers, like GBM themselves, find broaching the subject of anal sex difficult. Discussion of any sexual behavior may occur in less than 50% of meetings between HIV healthcare workers and their clients [18], and anal health is a specific and particularly challenging topic to discuss during clinical encounters [19, 20].

Healthcare worker training is one way to promote skills and innovate healthcare practices in the fight against HIV/AIDS [21], yet few interventions target, or rigorously evaluate, health workers’ comfort and capacity to address stigma toward anal sex [22, 23]. We sought to evaluate a stigma-reduction training program for healthcare workers in China. The primary intervention aim was to enhance provider comfort and capacity to discuss anal sex with clients, thereby improving HIV prevention and sexual health. Additionally, we sought to identify the mechanisms of action underlying any changes in provider behavior, in line with the scientific literature’s call to interrogate hypothesized pathways by which interventions operate to shift behavior [24, 25].

## Methods

To explore the potential impact of a training on anal health promotion activities and mechanisms of action among 51 healthcare workers, we conducted a pilot study using online surveys pre- and post-training as well as in-depth interviews post-training. The Institutional Review Board of the New York State Psychiatric Institute approved all procedures (Protocol #7676), as did Fudan University in China through an amendment to an open protocol from an ongoing three-year collaboration with the Aaron Diamond AIDS Research Center. Procedures are described in more detail elsewhere [26].

### Transparency and Openness

We report on our recruitment, sample size, measures, and analyses. We analyzed survey data in IBM SPSS Statistics 20 [27] and interview transcripts in Dedoose [28]. Requests for de-identified data may be submitted to the corresponding author.

### Local Context

The Good Participatory Practices (GPP) Program of the China AIDS Initiative was a three-year initiative to increase capacity of clinical research sites across six cities in China to engage GBM in HIV-related services and research, by fostering connections between clinicians, community-based organizations and companies providing HIV education, counseling, and testing services for GBM. Through ongoing stakeholder engagement activities at each clinical research site and mixed-methods research [29], the GPP program identified high perceived stigma and low perceived knowledge about anal sex by healthcare workers as barriers to GBM accessing HIV-related services and engaging in clinical research.

### Participants

#### Recruitment

Hospitals, community-based organizations, local health departments, and universities already engaged in the GPP Program identified members of their regional HIV workforce. The identified individuals included GBM trained as lay counselors in HIV testing programs, infectious disease physicians, and health department staff with epidemiology and other public health training – all members of the HIV healthcare workforce that served GBM. The GPP Program then used this list of roughly 80 individuals to send email invitations to a 2-day sexual health training to build knowledge about anal health and the links between anal health, pleasure, and HIV services. Those interested in participating in the training received a follow-up hyperlink to consent for study participation. The consent form detailed the pre- and post-intervention surveys and opportunity to be invited for a post-intervention, in-depth interview.

#### Sample

All 51 people who registered for the training consented to participate in research. The majority (*n* = 41, 80.4%) attended both days, with nine attending 50–75% and one person attending only 25% (a half-day). Thirty-six (70.6%) completed pre-training baseline measures and forty (78.4%) completed survey measures immediately after the training. Thirty-four participants (66.6%) completed both timepoints, allowing for statistical comparison. We conducted telephone interviews of all participants who expressed interest (*n* = 30, 59%); most of these (90%) had completed both training days. Two months after the training, 49 (96.1%) completed follow-up open-ended survey questions. Participants did not articulate reasons for their lack of participation in survey and telephone interview assessments. We found no differences in survey and interview participation, except that CDC workers and respondents from some cities were significantly more likely while hospital workers were significantly less likely to complete the baseline survey, and those who were employed full time were significantly more likely to participate in interviews. While significant, these differences are not interpretable.

### Procedures

Surveys were hosted through the China-based survey platform WenJuanXing. An anonymous identifier linked participants across timepoints, effectively blinding the analyst to the identity of survey respondents.

Hourlong telephone interviews were conducted in Mandarin by research assistants trained in qualitative inquiry by the first authors (BAK, KM). Interviewers attended the in-person workshops as observers and were known to the interviewees as staff involved in the GPP Program. Interviewees were provided with the equivalent of ~ US$30 compensation. Participants who completed final assessments were entered into a lottery to receive a ~ US$80 gift certificate. Telephone interviews were audio-recorded on a password-protected device and downloaded to a secure server, then the original was deleted. Research assistants completed a structured debriefing report after each interview to identify emergent themes. A third-party vendor transcribed and translated interviews, which the research assistants reviewed for accuracy and to redact identifiers.

### Training Intervention

The two-day, in-person training program, titled *Smarter Sex is the New Safer Sex: Anal Pleasure and Health 1.0*, was originally developed for quality improvement, not research, to equip the HIV workforce in the United States with knowledge and skills to broach conversations about anal pleasure and health with consumers of harm reduction services, inclusive of the diversity of sexual orientations and gender identities that engage in anal sex [23]. Although the program had not undergone scientific evaluation in the United States due to limited funding opportunities, the GPP project leader (KM) invited the program developer (BAK) to implement the training in China, and, at the developer’s request, they collaboratively developed a protocol to evaluate the training in China. For China, the training developer reviewed a detailed outline of the training objectives and activities with the GPP project leader (a researcher with decades of experience working in China), and a bilingual Chinese sex education trainer (YW), who advised translation of the materials into Mandarin without adaptation. The training was then co-facilitated by the developer and the Chinese sex education trainer, with the Chinese trainer providing live translation and adapting concepts to Chinese culture as needed based on participant feedback.

The program in China intended to stimulate HIV workers’ awareness of their own biases related to anal sex, increase their knowledge of basic anal physiology and sexual response, and enhance their communication skills to initiate discussion with clients who engage in anal sex, including but not limited to GBM. Exercises relied on the thoughts-feeling-behavior model from cognitive behavior therapy to reframe cognitive biases about anal sex and foster behavioral regulation in the face of anxiety about communication. Additional exercises taught the elicit-provide-elicit approach to information exchange from motivational interviewing [30]. Participants engaged in exposure-based activities, cognitive restructuring exercises, and skills-based practice sessions in multi-modal ways (i.e., individual reading, small group discussions, large group discussions, review sessions, and interactive roleplays). Trainers modeled communication skills. The overarching goal was to enable HIV workers to initiate and navigate candid dialogue with clients, ultimately promoting sexual health and well-being related to anal sex as well as client engagement in HIV services. Implementation outcomes of the workshop are described elsewhere [26].

## Assessments

### Data Collection

Data collection instruments were developed by the first three authors in English, then translated into Chinese by a bilingual research assistant. Another research assistant reviewed the Chinese version of the study instruments with two mono-lingual Chinese GBM and made adjustments to increase comprehensibility. Two other bilingual research assistants then back-translated into English and, following group discussion, adjusted and pilot tested the instruments.

We requested data from participants at three different timepoints: (A) an online survey one week prior to the training (July 2018), (B) in-depth telephone interviews one to two weeks after the training (August-September 2018), and (C) a follow-up online survey conducted two months after the training (October 2018). The second online survey included repeated measures from the first survey as well as open-ended questions to gather information about the impact of the training. As a pre-post quantitative evaluation, the pilot study aimed primarily to assess the acceptability, feasibility and appropriateness of the training and related study procedures and assessments. Despite the pilot study design not being designed nor statistically powered for inferential statistics, we assessed the preliminary impact of the training on mechanisms of action and anal health promotion activities.

### Quantitative Assessment

The cultural fit and relevance to the Chinese context were considered when generating items by drawing on the expertise of co-authors with combined decades of experience conducting research around HIV services and sexual health, and delivery of sexual health education in China.

#### Sociodemographics

At baseline, we used standard items utilized in ongoing GPP research in China to assess age; city of residence; education; ethnicity; gender identity; sexual orientation; student status; primary work duties; work setting; clientele (e.g., percentage of heterosexual men, gay/bisexual men); employment status; monthly income; and length of time in direct services.

#### Potential Impact on Frequency of Anal Health Promotion Activities

For anal health promotion target behaviors, we developed a 7-item measure of participants’ frequency conducting anal health promotion activities with GBM (e.g., “How often have you asked male clients about their anal sex practices?”). The measure indicated strong internal reliability (pre-training α = 0.86, post-training α = 0.90). Medical personnel responded to two additional questions about the frequency of screening activities (i.e., sexual history taking; anorectal examination). For all items, response options ranged from 1 (*Not at all*) to 5 (*Always*).

#### Potential Impact on Mechanisms of Action for Anal Health Promotion Activities

We also developed a 7-item measure of comfort conducting these same anal health promotion activities (e.g., “How comfortable are you asking GBM clients about their specific questions or concerns related to anal sexuality, apart from HIV/STIs?”). This measure also indicated strong internal reliability (α = 0.93 at both timepoints). Medical personnel additionally responded to two questions related to comfort conducting anal health screening activities (i.e., sexual history; anorectal exam). Response options ranged from 1 (*Not at all*) to 5 (*Extremely comfortable*). For additional mechanisms of action, we adapted the *Determinants of Implementation Behavior Questionnaire* (*DIBQ*). [31] Our 22-item scale (pre-training α = 0.82; post-training 0.86) assessed the impact of the training on seven of the most relevant constructs from the *Theoretical Domains Framework*, which posits a comprehensive set of mechanisms of action based on theory and expert opinion [32]. (See Supplementary Table 1 for additional domains that were assessed qualitatively.) Subscales largely reflected acceptable to excellent reliability between pre- and post-training: *knowledge* (α = 0.69; 0.87), *role responsibility* (α = 0.85; 0.94), self-efficacy (α = 0.79; 0.88), *organization* (α = 0.71; 0.74), and *positive emotions* (α = 0.74; 0.92). *Negative emotions* (α = 0.58; 0.84) and *skills* (α = 0.72; 0.49) demonstrated worse reliability at the pre- and post-training timepoints, respectively. Response categories ranged from 1 to 7 with variably defined anchors (e.g., *Very difficult* to *Very easy*; *Not worthwhile at all* to *Very worthwhile*; *Strongly disagree* to *Strongly agree*).

### Qualitative Assessment

Interviews were offered to all participants via an email invitation post-workshop. Guided by the articulation of mechanisms of action in the Theoretical Domains Framework [31, 32], we aimed to elicit participants’ reflections on the potential impact of the training on mechanisms that would need to change to promote discussion of anal pleasure and health. Additionally, the follow-up survey, timed two months post-workshop to allow for participants to have accrued exposure to clients for potential discussions about anal pleasure and health, included open-ended questions (e.g., “What from the training, if anything, helped [during conversations about anal sexuality with clients]?” and “How have you tailored, or how might you tailor, what you learned in the training to your GBM clients’ needs?”). An abbreviated qualitative interview guide is available in Supplementary Table 1.

### Analytic Plan

For quantitative analyses, we compared pre- and post-training survey responses using paired sample Wilcoxon Signed Rank Tests, among participants who completed assessments at both timepoints. We set statistical significance at *p* < .10. Our qualitative inquiry followed the same deductive approach in our thematic analysis as described in an earlier manuscript [26]. First, the interview transcripts were translated into English through a third-party vendor and reviewed by the research assistants for accuracy and to redact any identifying information. Then, three co-authors (BAK, KM, YW) conducted two initial learning periods involving a subset of nine transcripts and all two-month follow-up survey responses. Learning periods involved the extraction of power quotations and the development of individual diagrams of memorable content as well as project-level diagrams, from which the team identified themes related to the pre-specified TDF domains for further analysis and coding. Based on these themes and the qualitative inquiry prompts, we refined a codebook and then double-coded 10% of transcripts, resolving discrepancies by consensus. Each reviewer then applied the codebook and wrote memos on a third of the transcripts, analyzing how the content of each transcript contributed to or contradicted already developed themes or presented new insights. For this manuscript, an additional co-author (BL) joined two of the original coders (BAK, KM) to conduct two additional initial learning periods specific to the topic of effects of the training. This entailed reading all excerpts coded as “Training Effects”, and then the identification, discussion and revision of emergent themes from these quotations.

The qualitative analysis team included four people with varied backgrounds relative to Chinese culture: a US-based trainer with decades of HIV counseling and research experience in the US but no work in China; a US-based analyst involved only in data analysis; a US-based social scientist who lived in China for four years, spoke Mandarin, and had fifteen years of HIV research and community engagement experience in China; and a US-based Chinese citizen with four years as a sexual health educator in Chinese universities and two years coordinating HIV research and community engagement projects in China. The third and fourth analysts knew one-third of the training participants through their community engagement work. Therefore true blinding of the qualitative data was not realistic as even the redacted transcripts in some cases contained clues as to the identity of the interviewee. Since the team was aware of this, we took proactive steps to ensure that our analyses were grounded in the data, checking our subjective stances iteratively and returning to written transcripts to ensure that the identified themes were true to the data. In addition, for this analysis, we focused on transcript excerpts coded as “training effects,” which meant that the quotes were decontextualized from the full transcript, thereby rendering them less identifiable by analysts three and four.

## Results

### Sociodemographics

As seen in Table 1, the fifty-one training participants skewed toward younger age (M = 35, SD = 9). Most identified as cisgender male (86%) and Han ethnicity (90%). They reported high education, half with college degrees and another 37% with graduate degrees. Modal income was ~ US750-1500/month. Half (47%) identified as gay/homosexual and 14% as bisexual. Participants reported that 70% (SD = 31%) of their client populations identified as GBM. About half conducted HIV counseling and testing, 20% primarily public health interventions, and 14% HIV treatment and 8% STI treatment as medical doctors. Modal career length was 5–10 years (29%), with fewer reporting 2–5 years (22%) and 10 years or more (24%).

**Table 1:** Demographic characteristics of Chinese health workers (*N* = 51)

Variable	*n*	(%)	Mean	(SD)
Age (in years)			34.53	8.58
Education				
Middle school	1	2.0		
High school or equivalent	6	11.8		
College	25	49.0		
Above college	19	37.3		
Han ethnicity	46	90.2		
Cisgender male	44	86.3		
Sexual orientation				
Gay/homosexual	24	47.1		
Straight/heterosexual	20	39.2		
Bisexual	7	13.7		
Client population (percentage)				
Gay/bisexual men			69.9	31.0
Heterosexual men			15.2	19.7
Heterosexual women			8.8	13.4
Lesbian/bisexual women			2.2	6.4
Transgender men or women			2.0	3.8
Primary work duties				
HIV counseling & testing	26	51.0		
Public health interventions	10	19.6		
HIV treatment	7	13.7		
STI treatment	4	7.8		
Other	4	7.8		
Work setting				
CBO	27	52.9		
Hospital	16	31.4		
CDC (Chinese Center for Disease Control and Prevention)	14	27.5		
STI Clinic	8	15.7		
Additional	3	5.9		
Employment				
Full-time	43	84.3		
Part-time	6	11.8		
Other	2	3.9		
Monthly income				
No income	3	5.9		
2000 RMB – 3999 RMB	6	11.8		
3000 RMB – 4999 RMB	16	31.4		
5000 RMB – 9999 RMB	19	37.3		
10,000 RMB – 19,999 RMB	5	9.8		
20,000 RMB – 29,999 RMB	2	3.9		
Career length				
Less than 1 year	8	15.7		
1–2 years	5	9.8		
2–5 years	11	21.6		
5–10 years	15	29.4		
10–20 years	9	17.6		
20 or more years	3	5.9		

### Quantitative Findings

#### Potential Impact on Frequency of Anal Health Promotion Activities

As seen in Table 2, post training, participants reported being significantly more likely to engage in three anal health promotion activities: (A) initiating conversation about anal health, (B) responding to concerns about anal health, and (C) responding to concerns about anal sexuality more generally. Frequencies increased between 7 and 8% of the scale range (*p* = .06-0.08). Other anal health promotion activities also increased in the direction of change, but not significantly.

**Table 2:** Frequency and comfort with anal health promotion activities from baseline to follow-up among Chinese health workers (*N* = 34)

		Baseline	Follow-Up		
Variable	*n*	*Mean* (*SD*)	*Mean* (*SD*)	*Mean Difference* *(% change)*	*Z* (*p*-value)
Frequency of activities*					
Asked male clients about their sexual orientation.	34	3.79 (1.43)	3.94 (1.28)	0.15 (3%)	-0.81 (0.42)
Asked MSM clients about their anal sex practices.	34	4.03 (1.29)	4.12 (1.23)	0.09 (2%)	-0.50 (0.61)
Discussed options for HIV/STI prevention with MSM clients.	34	4.29 (0.87)	4.18 (1.03)	-0.12 (-2%)	-0.86 (0.39)
Initiated a conversation with MSM about anal health, apart from infectious disease.	34	2.50 (1.26)	2.85 (1.05)	0.35 (7%)	-1.91 (0.06)
Responded to anal health concerns with your MSM clients, apart from infectious disease.	34	2.79 (1.27)	3.18 (1.03)	0.38 (8%)	-1.78 (0.08)
Asked MSM clients about their specific questions or concerns related to anal sexuality, apart from HIV/STIs.	34	2.62 (1.16)	2.82 (1.06)	0.21 (4%)	-1.38 (0.17)
Responded to specific questions or concerns about anal sexuality from MSM clients.	34	2.71 (1.17)	3.09 (1.06)	0.38 (8%)	-1.79 (0.07)
Medical personnel only					
Sexual history with MSM clients	5	4.00 (1.73)	4.20 (0.84)	0.20 (4%)	0.00 (1.00)
Anorectal exam with MSM clients	5	2.40 (1.34)	2.60 (1.52)	0.20 (4%)	-0.38 (0.71)
Comfort with activities**					
Asking male clients about their sexual orientation.	34	4.50 (0.75)	4.71 (0.58)	0.21 (4%)	-1.81 (0.07)
Asking MSM clients about their anal sex practices.	34	4.29 (0.84)	4.71 (0.58)	0.41 (8%)	-3.73 (0.01)
Discussing options for HIV/STI prevention with MSM clients.	34	4.59 (0.82)	4.76 (0.50)	0.18 (4%)	-1.19 (0.24)
Initiating a conversation with MSM about anal health, apart from infectious disease.	34	3.88 (1.20)	4.44 (0.82)	0.56 (11%)	-2.84 (0.01)
Responding to anal health concerns with your MSM clients, apart from infectious disease.	34	3.91 (1.16)	4.56 (0.61)	0.65 (13%)	-2.84 (0.01)
Asking MSM clients about their specific questions or concerns related to anal sexuality, apart from HIV/STIs.	34	3.91 (1.24)	4.32 (0.81)	0.41 (8%)	-2.21 (0.03)
Responding to specific questions or concerns about anal sexuality from MSM clients.	34	4.00 (1.13)	4.44 (0.70)	0.44 (9%)	-2.52 (0.01)
Medical personnel only					
Sexual history with MSM clients	5	3.60 (1.34)	4.60 (0.55)	1.00 (20%)	-1.63 (0.10)
Anorectal exam with MSM clients	5	3.20 (1.48)	3.40 (1.14)	0.20 (4%)	-0.58 (0.56)

All item Likert responses ranged from 1 to 5, with higher scores indicating greater positive valence

*1 Not at all, 2 Sometimes, 3 Often, 4 Very Often, 5 Always

**1 Not at all comfortable, 2 Slightly comfortable, 3 Moderately comfortable, 4 Very comfortable, 5 Extremely comfortable

Among the five clinical personnel who completed pre- and post-training measures, there was a 4% increase in the frequency of their conduct of a sexual history and of their conduct of anorectal examinations, but neither were significant increases.

### Potential Impact on Mechanisms of Action

#### Comfort Conducting Anal Health Promotion Activities

Also in Table 2, participants reported increases, ranging from 4 to 13%, in comfort asking about sexual orientation (*p* = .07); asking about anal sex practices (*p* = .01); initiating conversation about anal health apart from infectious disease (*p* = .01); responding to anal health concerns apart from infectious disease (*p* = .01); asking about client questions or concerns related to anal sexuality apart from HIV/STIs (*p* = .03); and responding to client questions or concerns about anal sexuality (*p* = .01). Medical personnel reported a nearly significant 20% increase in comfort conducting a sexual history (*p* = .10).

#### Determinants of Implementation Behavior

As seen in Table 3, mechanisms of action related to discussion of anal sexuality (*knowledge*,* skills*,* role responsibility*,* self-efficacy*,* organizational acceptability*, and *positive emotions*) increased significantly from baseline to follow-up, from 5% − 36% of the scale range (all *p* < .05). Overall, subscales for *knowledge* and *skills* shifted the most (28% and 36%, respectively), driven by greater reported knowledge about anal physiology and pleasure as well as greater reported skills to talk about and respond to questions about anal health. Small increases in *role responsibility* (5%) were driven by a shift in responsibility to help clients feel more comfortable disclosing anal sex during a clinical encounter (8%). A small increase (4%) was also reported for *responsibility to improve one’s own comfort discussing anal sexuality* (*p* = .08). *Self-efficacy* was driven by increased confidence talking about anal health even when clients are not motivated (13%). Likewise, *organizational acceptance* to discuss anal health with GBM was driven by support from management (9%, *p* = .01); there was no significant change in acceptance within one’s workplace more generally (4%, *p* = .13). In terms of participants’ general feelings toward delivering services to GBM, not specifically related to anal health, “ease” and “comfort” drove an overall significant increase in *positive emotions* (8%, *p* < .005), with no significant changes in *negative emotions* (*p* = .45).

**Table 3:** Determinants of implementation behavior questionnaire about training effects among Chinese health workers (*n* = 34)

	Baseline	Follow-Up		
	*n* = 34	*n* = 34		
Variable	Mean (SD)	Mean (SD)	MD (% change)	t (*p*-value)
Knowledge	4.25 (1.31)	6.19 (0.79)	1.94 (28%)	−9.17 (0.00)
I know basic information about anal physiology and sources of pleasure.	3.79 (1.65)	6.24 (0.82)	2.44 (35%)	−7.93 (0.00)
I am familiar with why MSM have anal sex.	5.18 (1.64)	6.18 (0.94)	1.00 (14%)	−3.57 (0.00)
I am aware of how to communicate basic information about anal physiology and pleasure with MSM clients.	3.76 (1.71)	6.15 (0.89)	2.38 (34%)	−9.03 (0.00)
Skills	3.47 (1.41)	5.96 (0.84)	2.49 (36%)	−10.28 (0.00)
I have been trained to talk about anal health with MSM clients.	2.74 (1.58)	6.03 (1.22)	3.29 (47%)	−9.16 (0.00)
I have the skills to discuss anal health with MSM clients.	4.18 (1.98)	5.91 (1.14)	1.74 (25%)	−5.98 (0.00)
I have practiced ways to respond to MSM questions about anal health.	3.50 (1.71)	5.94 (1.23)	2.44 (35%)	−7.52 (0.00)
Role	5.93 (1.02)	6.31 (0.82)	0.38 (5%)	−2.65 (0.01)
Being able to talk about anal health with MSM is a part of my work as an HIV worker.	5.94 (1.10)	6.21 (0.95)	0.26 (4%)	−1.36 (0.18)
As a health worker, it is my job to increase my own comfort discussing anal sexuality with MSM clients.	6.09 (1.14)	6.38 (0.82)	0.29 (4%)	−1.83 (0.08)
It is my responsibility as a health worker to help MSM feel more comfortable disclosing about anal sex.	5.76 (1.26)	6.35 (0.85)	0.59 (8%)	−3.11 (0.00)
Self-efficacy: I am confident that I can…	5.18 (1.24)	5.80 (0.99)	0.63 (9%)	−3.80 (0.00)
Talk about anal health with MSM clients even when they are not motivated.	4.82 (1.62)	5.71 (1.22)	0.88 (13%)	−3.45 (0.00)
Talk about anal health with MSM clients even when other health workers with whom I work do not do this.	5.50 (1.29)	6.06 (0.95)	0.56 (8%)	−3.10 (0.00)
Discuss anal health with MSM clients even when there is little time.	5.21 (1.51)	5.65 (1.12)	0.44 (6%)	−1.79 (0.08)
Organizational Acceptance	5.66 (1.28)	6.13 (0.96)	0.47 (7%)	−2.43 (0.02)
Discussing anal health with clients is acceptable within my workplace.	5.94 (1.39)	6.24 (0.96)	0.29 (4%)	−1.54 (0.13)
The management of the organization I work in is supportive of addressing anal health with MSM.	5.38 (1.52)	6.03 (1.19)	0.65 (9%)	−2.67 (0.01)
Positive emotions: When I deliver services to MSM…	5.66 (1.05)	6.23 (0.84)	0.57 (8%)	−3.81 (0.00)
I feel natural.	6.09 (1.16)	6.35 (0.85)	0.26 (4%)	−1.66 (0.11)
I feel at ease.	5.79 (1.37)	6.38 (0.82)	0.59 (8%)	−2.84 (0.01)
I feel comfortable.	5.21 (1.65)	6.24 (0.99)	1.03 (15%)	−3.83 (0.00)
I feel proud.	5.56 (1.40)	5.94 (1.07)	0.38 (5%)	−1.65 (0.11)
Negative emotions: When I deliver services to MSM…	2.80 (1.08)	2.62 (1.40)	−0.18 ( −3%)	0.76 (0.45)
I feel nervous.	2.50 (1.50)	2.50 (1.80)	0.00 (0%)	0.00 (1.00)
I feel awkward.	2.35 (1.67)	2.32 (1.66)	−0.03 (0%)	0.01 (0.94)
I worry I’m going to say or do the wrong thing.	3.74 (1.76)	3.38 (1.89)	−0.35 ( −5%)	1.12 (0.27)
I feel I don’t know what to do.	2.62 (1.60)	2.26 (1.42)	−0.35 ( −5%)	0.98 (0.34)

All item Likert responses ranged from 1-7, with higher scores indicating greater positive valence

1 Strongly disagree, 2 Disagree, 3 Somewhat disagree, 4 Neither agree nor disagree, 5 Somewhat agree, 6 Agree, 7 Strongly agree

### Qualitative Findings

Drawing from 30 telephone interviews and 49 follow up text entry responses, six themes are explicated below and are supported with illustrative quotations in Table 4.

**Table 4:** Themes and illustrative quotations among Chinese health workers (*n* = 30 interviews, *n* = 49 text entry survey responses)

Theme 1: Information about pleasure increased empathy, weakened stereotypes, and reframed anal sex from a “deviant” to a “normal” sexual behavior
*1.1 Understanding and empathy*
“If you don’t understand their pleasure, you can’t understand why they want to have anal sex… [the training] was very helpful and quite new, and covered some blind areas in our knowledge. We didn’t understand why these people would like to have anal sex. We understood better after the training.” (IDI-45, male, heterosexual, STI doctor) “After I learned the knowledge of anal physiology and understood why anal sex is pleasant, I accepted the feeling of my clients naturally and became more empathetic.” (IDI-39, male, heterosexual, HIV counselor)
*1.2 Normalizing anal sex*
“Can there be more Chinese women than Chinese men who have anal sex? During the training, my feeling was, what?! There are so many women having anal sex, more than men! I’ve never heard this. I had no idea, to be frank with you. So, I’ve been thinking, that if we receive and examine female patients, we need to check their anal area.” (IDI-41, male, heterosexual, STI doctor) “[The trainer] talked about some statistics released by some bureau in the US. It let me know that, wow, it turned out that women are perhaps more active in having anal sex than men. Previously, I always thought anal sex is more common among homosexuals. I didn’t expect that anal sex also happens among heterosexual men and women, that they can get as much pleasure as men who have sex with men. I think this is important information which changed my mind to a great extent.” (IDI-38, male, bisexual, other public health duties)

Theme 2. Participants recognized sexual health and pleasure as appropriate to discuss with clients, but some disagreed that promoting pleasure would benefit client health.
*2.1 Recognizing sexual health and pleasure as appropriate topics to discuss with clients*
“I think this approach – starting from the topic of sexual pleasure – could be helpful for disease control and prevention. There could be different outcomes if you start from the topic of sexual pleasure and then move to disease prevention. The reality is, our clients are more likely focused on sexual pleasure. They may be reluctant to engage with us when we begin from disease. So, the new approach I am considering is not to emphasize AIDS or STDs when a patient comes to us, not to start with things about how they [MSM] should behave from a sense of responsibility…. The outcome of our practice hasn’t been so good after so many years of experience. But if you handle the things in another way, using the topics that he is interested in or the points of his focus to introduce the behavior change, this could achieve disease prevention and control. I think this could be a very good approach.” (IDI-32, male, heterosexual, STI doctor) “I may simply tell them that it’s normal that they get pleasure in this [anal sex]. As a doctor, if I could give him this information or hint, that’s enough. It may work pretty well.” (IDI-39, male, heterosexual, HIV counselor)
*2.2 Disagreeing that promoting anal pleasure would benefit their clients’ health*
“Since many people from CBOs were present, I worried [that the focus on pleasure] might give them a misunderstanding: ‘After I knew the correct way of anal sex and how to relax, I could reduce the chances of bleeding. As a result, it became unnecessary to use condoms.’ I worried that might be the case, which would lead to the reduced rate of condom use. That’s why I felt it should have been further emphasized.” (IDI-47, female, heterosexual, CDC staff) “So far, I haven’t met anyone who insists on not wearing condoms because doing so makes sex not pleasurable. […] I feel it’s unnecessary to speak about anal pleasure with people who wear condoms. From what I understand, people wearing condoms don’t have any issues with experiencing pleasure. On the other hand, those who don’t wear condoms speak about pleasurable or non-pleasurable sex. Those who wear condoms don’t think pleasure is important. For them safety is more important.” (IDI-50, male, gay, HIV counselor)

Theme 3. Detailed, practical information and skills to communicate about anatomy, physiology, and pleasure increased provider comfort and confidence, but a one-time training was not sufficient to increase or sustain these changes among all participants
*3.1 Increasing provider comfort and confidence*
“I think regarding the knowledge taught [during the training], there are many aspects that we knew already and told others about. However [in the past] there was no way for me to talk about how to have pleasure or how to improve pleasure during anal sex. Based on my knowledge, basically nobody every mentioned it. It is like this: I can tell clients what *not* to do, but I had no way to tell them what t*o do*. [After this training] I can take more initiative now since I have things to offer them.” (IDI-46, male, bisexual, CDC staff) “I felt more confident to deliver anal sex knowledge to my clients, so I have better communication with them. … After training, I can use the skills to communicate smoothly when the client is shy or worried.” (IDI-37, male, gay, HIV counselor) “Everytime I or my clients felt embarrassed during the consulting or I did not express myself clearly when answering my clients’ questions, the anal physiology knowledge I learned from the training would save the atmosphere of the conversation between me and my clients.” (IDI-49, male, gay, HIV counselor)
*3.2 A one-time training was not sufficient to increase or sustain comfort and confidence among all participants*
“I need extra support as reading the materials alone can’t help me to understand or master the knowledge. … We cannot just answer those questions casually or pretend to answer the questions. We need to be professional in our answers. If they come to the counseling, they come to seek a very accurate answer. In case that we are not completely familiar with the topic, the respondents may have the impression that the counselors don’t even understand the topic, how to trust them to discuss about the topic? They may be reluctant to come again.” (IDI-44, male, bisexual, HIV counselor) “[S]ince there other members of our CBO where were also there, I asked them their feelings about the training. One person told me that he thought the content was new and it was the first time for him to hear about the topic. For him, if clients asked him about [anal sex] in the future, he can answer with more ease. Another volunteer told me, however, that since some things in the training differed from what he previously imagined, it perhaps created some confusion for him.” (IDI-48, male, gay, HIV counselor)

Theme 4. Awareness of the lack of knowledge about anal pleasure and health increased intentions to discuss the subject with clients and colleagues, yet some continued to harbor beliefs about negative consequences if they were to broach the subject
*4.1 Recognizing lack of knowledge*
“A colleague of mine and I were planning to organize an event about what was taught in the training [in our own community]. Because I feel there is a lack of knowledge on this topic within our community. If the event can successfully happen, it could at least help a large number of people gain an awareness about correct anal sex practices and anal health.” (IDI-31, male, gay, CDC staff) “As far as I know, from my chatting with other participants, they shared my feelings. They told me, ‘Wow, this knowledge is something we never knew before.’ Something that we believed to be in one way turned out to be another way. The more we learnt, the more we realized that we still don’t know. We all said it was very good and it opened our eyes.” (IDI-38, male, bisexual, other public health duties)
*4.2 Increasing intentions to discuss anal pleasure*
“In the past, when people asked me something about this [anal sex], I either ignored them or felt quite embarrassed. I think I can now discuss this with my clients directly and positively, without any evasion or fudging….from my personal perspective, I think I better understand the topic of anal health. I think there are many beneficial things that come with this better understanding and I’d like to share these beneficial things with more people.” (IDI-44, male, gay, HIV counselor) “There is a high probability that I will organize a sharing event on this topic, which will take place not only once or twice, but many times. I really think that we need to talk about this. During the training, almost all of us said it was the first time they got in touch with content of anal sex. So, if we organize this kind of sharing event, if we can make that happen, it could be the first time for the gay community in City X to learn this information…That’s why I want to frequently organize such sharing events.” (IDI-43, male, gay, other public health duties)
*4.3 Beliefs about negative consequences of broaching anal pleasure with clients*
“What could happen [if I talk about these topics with MSM clients]? I think the most probable situation is that he may feel embarrassed, but I am not. He could feel embarrassed when he talks about this with me. This is the first possibility. The second scenario is he doesn’t think that the information I provide is accurate. The other is… and it’s one reason that I don’t really want to talk about this topic, I fear that they will feel that I’m harassing them…” (IDI-48, male, gay, HIV counselor) “I’m just worried that [a client] will ask me a question and I may forget an answer learnt in the training. Or I may feel very embarrassed if he asked a question that I don’t quite know. It would be rather awkward. Then I couldn’t talk my out of it. Then, that would not be good.” (IDI-36, male, gay, HIV counselor) “I don’t think I would raise this topic first normally … especially for these MSM since it’s quite sensitive and my identity is not suitable to raise this topic abruptly. It may be offensive I think. But in a casual talk, I may mention to them that I recently attended a training on the subject and I learnt something. I may ask if he is interested. In chatting, I may mention things related to this area to them. [Oh, like that] But definitely I won’t talk in a very direct way like ‘Do you want to increase the quality of your sexual life?’ I will never do that since I don’t think it’s polite to do so. And they would also consider that I’m not of the same sex as them, this is also very important. And on the other hand, my job is not a counselor for sexual health, I’m not working in an NGO [non-governmental organization], but I’m working in a hospital. So, they have totally different impression about me.” (IDI-1420, female, heterosexual, HIV doctor)

Theme 5. Participants shared information about anal pleasure and health that they learned from the training with clients.
*5.1 Disseminating information*
“After the training, I added anal health questions to my clients during the counseling and the test. Most clients show their interests to this topic. Someone told me, he learned this kind of knowledge from Baidu, but it’s not accurate on Baidu. The information from me is comprehensive. He now knows the structure, health and sexual pleasure of each body part and it’s very helpful.” (IDI-40, male, heterosexual, HIV counselor) “I felt comfortable and easy during the conversation [talking with a client about anal sex], because I could use not only my perspective and experience, but also the theoretical knowledge learned in the training to answer their questions in a straightforward manner” (IDI-44, male, gay, HIV counselor)
*5.2 Sharing with other health workers was not always acceptable*
“[W]hen you asked about embarrassment, when I trained other staff in our department, it was true that some people could not accept this. After I returned to work I trained other members of my department. I copied the promotional materials and gave one copy to each of them so that they could learn and they could discuss with me if they wanted, right? It turned out, most of them couldn’t accept that. … I think it was probably because our identities are different. Maybe because I’m a doctor while others are [nurses]… maybe it’s because I’m a male while all the others are females. That was the first of my two major feelings since I returned [from the training]. The second was, among all the people you recruited for training, not all of them could accept the training… the knowledge about pleasure in anal sex. Because people all have a fixed mind, and they will resist this kind of content. Not everyone who went to the training would feel as I did, which was “I’ve learned so much.” (IDI-41, female, heterosexual, STI doctor) Participant: “Let’s face it, there is no way to copy the two days’ training. I compressed it into two hours. I briefly trained them with what I learnt. In this process, I can feel people felt embarrassed.” Interviewer: “They were embarrassed. Did you try to relieve the embarrassment?” Participant: “I tried, but I didn’t spend much time on it.” Interviewer: “Okay. So, your main purpose was to pass on the content to them, right?” Participant: “Yes, and they all have many years of working experience. Probably this embarrassment came from the fact that I didn’t help them get desensitized enough. And I didn’t conduct the ice breaking activities well. Or it was due to my training skills. But, what should I say? They have the capability to accept this. It will just take more time for them to accept.” (IDI-34, female, heterosexual, CDC staff)

Theme 6. Gay and bisexual participants reported that the training had a positive impact on their own relationship to anal pleasure and health
*6.1 Gay and bisexual men reported an improved understanding of pleasure and how to prevent harm during anal sex.*
“Those who have traveled with me and the ones who were close to me during the training all thought they benefited a lot from it. … They thought that they were able to know their own body better through the explanation of physiological knowledge, and how to get pleasure from the body. It enriched people’s understanding of the anus.” (IDI-35, male, bisexual, HIV counselor) “Many people, myself included, thought that when encountering a bigger penis, you might bleed and it is perfectly normal. Yes. Although we used more lubricants, we weren’t very clear about the differences of internal and external anal sphincters. In the past we knew that being relaxed can help, but even after being relaxed, it still bled and hurt. That’s why we thought those might be normal phenomenon in anal sex. But, the training changes some of these previous beliefs.” (IDI-33, male, gay, CDC staff)
*6.2 Gay and bisexual men reported that the training improved how they viewed their relationship to anal sex*.
“I know [the training] influenced me positively. In my previous sex life, I wasn’t able to enjoy sexual pleasure. More often, I wanted the other half to have the pleasure, so I must get it done even if I have to endure the pain. However, after the training, I learned there are so many ways. I could do it this way to make myself more comfortable. I can try that. The training increased my interest in anal sex. It’s not like before that I took it passively, instead, I can actively look for the right spot. Of course, a harmonious sex life will make romantic life more harmonious.” (IDI-43, male, gay, other public health duties) “In fact, yes, during anal sex in the past, it might feel painful or even agonizing. After the training, in fact, when I talked with them [sex partners], it was kind of like I had subconsciously gained knowledge that I could apply… by altering my positions and adding more lube, plus being less nervous than before, it feels probably less agonizing. Although [I] tried [having anal sex] before, there was always a little trauma, whereby when something foreign penetrates my body, I would feel nervous for no reason or couldn’t relax. After this training, [I] felt the process of [having anal sex] seems to have become smoother. (IDI-51, male, gay, HIV counselor)

*IDI* in-depth interview; identification numbers have been recoded to further protect confidentiality; *CDC* Chinese Center for Disease Control and Prevention

#### Theme 1

Information about pleasure increased empathy, weakened stereotypes, and reframed anal sex from a “deviant” to a “normal” sexual behavior.

1.1 **Understanding and empathy.** Increased knowledge, including the physiological basis for anal pleasure, allowed participants to better understand why their clients chose to have anal sex. This was particularly true of doctors and counselors who did not identify as GBM. Understanding that anal sex could be pleasurable due to physical stimuli, regardless of gender identity or biological sex, was also seen as critical to decreasing stigma by increasing understanding and empathy.
1.2 **Weakening stereotypes.** Many interviewees were surprised to learn that anal sex can be painless. Knowledge about the ways people prepare for penetration, including the liberal use of lubrication, using foreplay, and going slowly, were received as novel and useful. This new knowledge rectified misconceptions and weakened the stereotypical belief that people just have to tolerate pain and discomfort during anal sex.
1.3 **Normalizing anal sex.** Participants reported that learning about the prevalence of anal sex among women and heterosexual men reframed anal sex as a normal behavior. This stimulated participants to recognize, some for the first time, the relevance of anal health promotion activities with women in particular. Some expressed doubt that the prevalence of anal sex reported in the US and in Western Europe would be similar in China, but they nonetheless accepted that the data suggested a need to consider anal health beyond just GBM.

#### Theme 2

 Participants recognized sexual health and pleasure as appropriate to discuss with clients, but some disagreed that promoting pleasure would benefit client health.

2.1 **Recognizing sexual health and pleasure as appropriate topics to discuss with clients.** Some medical providers articulated how client education about anal pleasure could positively impact their clients’ behavior, including to reduce the risk of infectious disease. They cited specific information from the training (e.g., how to reduce physical and emotional discomfort, adding lubricant, options for lubricant, changing positions, breathing techniques, relaxing during bowel movements to reduce the potential for hemorrhoids and subsequent bleeding during sex) that they deemed appropriate and beneficial to share with their clients.
2.2 **Disagreeing that promoting anal pleasure would benefit their clients’ health.** While some participants reported increased motivation to broach anal pleasure and health with their clients, others seemed confused by the “sex positive” premise of the training. Two participants expressed strong negative beliefs about discussing anal pleasure. One viewed pleasure as a topic to raise only when clients refuse to wear condoms, and the other worried that a focus on pleasure would come at the expense of clients’ use of condoms.

#### Theme 3

 Detailed, practical information and skills to communicate about anatomy, physiology, and pleasure increased provider comfort and confidence, but a one-time training was not sufficient to increase or sustain these changes among all participants.

3.1 **Increasing provider comfort and confidence.** Doctors and healthcare workers who did not self-identify as GBM reported that better understanding why people enjoy anal sex, coupled with useful tips that they could share with clients to improve their sexual experience, increased their own comfort and confidence entering into client interactions. Lay health workers and counselors who identified as GBM expressed that the professional and technical nature of the information that they gained imbued them with a certain degree of expertise and thus authoritativeness to bring to encounters with clients. Participants appreciated that skills taught and practiced during the training (e.g., Elicit-Provide-Elicit from motivational interviewing) could be used to improve their health communication. They referred to increased confidence after learning skills and using them to help clients feel more at ease discussing anal sex.
3.2  **A one-time training was not sufficient to increase or sustain comfort and confidence among all participants.** Before they would be ready to initiate the topic with clients, some participants wanted to be more prepared. They mentioned needing to sustain gains in knowledge and comfort through future reinforcement, whether via review of training materials or additional learning resources. Others remained too confused about the content on anatomy, physiology and sexual response to feel confident sharing what they had learned.

#### Theme 4

 Awareness of the lack of knowledge about anal pleasure and health increased intentions to discuss the subject with clients and colleagues, yet some continued to harbor beliefs about negative consequences if they were to broach the subject.

4.1 **Recognizing lack of knowledge.** Training participants reported that the information about anal pleasure and health was new not only for providers but also for community members. They further reported a need to address this information gap in their communities.
4.2 **Increasing intentions to discuss anal pleasure.** Participants generally considered the information on anal pleasure and health to be important enough that they intended to share the content beyond the training, including to friends and to colleagues during in-house training. Respondents had profound realizations that sexual health promotion for GBM could extend beyond condom use and sexual positioning (i.e., topping, bottoming) to include discussions about pleasure. They considered this achievable through the promotion of an accurate understanding of anal anatomy and physiology.
4.3 **Beliefs about negative consequences of broaching anal pleasure with clients.** Some participants continued to believe that broaching the subject of anal pleasure with clients would feel embarrassing (for both clients and the provider), risk misinterpretation of the provider’s anal health promotion actions as sexual harassment, and cause some clients to doubt the provider’s credibility. One participant reported that the social context determined the potential for these negative consequences. For example, this participant maintained that clinical care was an inappropriate context to inquire about the quality of a client’s sex life, despite the fact that broaching pleasure and health with clients was the target of the training.

#### Theme 5

 Participants shared information about anal pleasure and health that they learned from the training with clients.

5.1 Disseminating information. Those who shared information with other community practitioners and gay and bisexual friends and clients described: (A) active and passive methods (e.g., posting on WeChat, holding a staff in-service); (B) sharing at the individual level and at community or organizational levels (e.g., passing information to a friend; developing health promotion materials like a brochure); and (C) formal and informal communications (e.g., casual debriefing of the training, educational sessions with the gay community).
5.2 **Sharing with other health workers was not always acceptable.** Some audiences were not receptive or did not think the information was credible. Participants did not always recall information accurately or frame the content in a sex positive fashion and some shared the content in ways that had unintended negative effects, which one participant credited to lacking the training trainer’s pedagogical skills. This suggested a limit to the impact of the intervention on promotion of anal health discussions beyond the participants who attended the training. Some participants anticipated that clients would be more receptive to the information if delivered in an alternative to a traditional provider-client 1:1 interaction.

#### Theme 6

 Gay and bisexual participants reported that the training had a positive impact on their own relationship to anal pleasure and health.

6.1 **Gay and bisexual men reported an improved understanding of pleasure and how to prevent harm during anal sex.** Four participants reported that the training, by providing accurate information and practical recommendations, changed how they envisioned approaching their future sexual encounters. They remarked that they had not known that anal sex could be painless and enjoyable, noting breathing practices, body positioning, douching, lubricants, and prostate stimulation as takeaways for a more pleasurable experience.
6.2 **Gay and bisexual men reported that the training improved how they viewed their relationship to anal sex.** GBM participants described past negative, non-pleasurable experiences and nervousness during sex. They expressed that their newfound knowledge about pleasure had the potential to improve their assertiveness, relationships, and ability to relax rather than remain anxious during sexual encounters.

## Discussion

Our analysis revealed that for most participants the theory-informed training activities operated as we hypothesized, to enhance mechanisms of action that could increase the initiation and discussion of anal pleasure and health within the context of HIV services. We found preliminary evidence that in the high-stigma context of China, the training normalized anal sex, reduced stereotyping, improved empathy, and shifted participants’ sense of professional responsibility to broach the topic with clients. Training participants likewise reported significant increases in their comfort, confidence and skills to initiate these discussions. We also found evidence of mechanisms of action that were not measured in the survey instruments (e.g. beliefs about consequences). Additionally, unsolicited by interviewers, GBM participants shared that the training improved their personal understanding of how to have anal sex in more healthful ways, suggesting that the content is likely to benefit GBM clients. However, among a minority of participants the training’s activities insufficiently activated mechanisms of action. This prompted us to analyze how the training did and did not operate on intended mechanisms and which additional behavior change techniques, if incorporated into the intervention, could increase its impact. We based our analysis on the Behaviour Change Wheel [42], the framework which forms the transtheoretical basis for the Theoretical Domains Framework and therefore the mechanisms assessed in our surveys and interviews. The BCW also offers guidance about related behavior change techniques that may shift specific mechanisms [43, 44]. This led to three key findings that apply specifically to urban Chinese contexts, but with potential applicability to other high-stigma contexts.

### Key Finding 1

Credible information led to knowledge that disrupted stigma processes.

Training activities that shaped knowledge by providing credible, scientific information about the prevalence of anal sex and anatomical sources of anal pleasure across populations (not just GBM) motivated participants to discuss the subject with their clients. Learning about the lifetime prevalence of anal sex among women was particularly surprising and salient for participants. The inclusion of this information was intended to disrupt stigma practices, like labeling and stereotyping [7, 33], by expanding mental models about who has anal sex. Training participants noted that the surprisingly high prevalence of anal sex among women in the US was unlikely to be true in China. However, a more recently published longitudinal study of sexual practices within heterosexual marriages between 2000 and 2015 reported pooled prevalence of anal sex of 7%, characterized by an increasing upward trend and reaching 10% in 2016 [47]. These findings suggest that anal sex is more common among heterosexual women in China than our HIV worker participants assumed, reinforcing the importance of sharing accurate, population-level data to challenge stereotypes and promote more inclusive, stigma-free sexual health conversations.

The emphasis on pleasure also provided counterstereotypic, positive information [34]. Rather than reinforce stereotypes (e.g., that GBM seek to emulate femininity by being penetrated; that anal sex must always be painful) participants learned that pleasurable sexual responses relate to nearly universal human anatomy [34–39]. This may have somewhat disrupted the stigma process of separating “us” vs. “them” [7], a mechanism not assessed in our study but documented in other stigma literature and interventions, including related to anal sex [33, 40, 41].

### Key Finding 2

Beliefs about negative consequences could be mitigated by introducing behavior change techniques related to natural consequences, comparison of outcomes, and incentivization.

Even though most participants reported feeling more motivated to broach discussions about anal pleasure and health with their clients, some remained preoccupied by beliefs about the negative consequences of being the first to address the topic during a clinical encounter. These participants felt no more capable or motivated to bring up the topic of pleasure, believing that the consequences could be counterproductive. The *Behaviour Change Wheel* [42] offers guidance about behavior change techniques that may shift these beliefs about consequences [43, 44]. These include natural consequences (e.g., the provision of information about the social, emotional and health benefits of discussing pleasure or the consequences of not addressing pleasure); comparisons of outcomes (e.g., comparative imagining of the positive outcome of initiating discussion vs. waiting passively for clients to broach the subject); and incentivization (e.g., informing or actually rewarding efforts to initiate discussion) [45]. We should also note that the training relied on incidental disclosure of client experiences by fellow participants who engage in anal sex; the introduction of narratives from clients about their experiences of stigma and lack of information [4, 5, 9, 13, 46, 47] may further mitigate beliefs about negative consequences by emphasizing the benefits of proactive discussion.

### Key Finding 3

Adding guidance on how to start a conversation about anal pleasure and health as well as providing client-facing materials could shift environmental resources and context in ways that enable health workers to initiate discussions.

In addition to more targeted exercises within the training, participants relayed a clear message that augmenting the training materials with physical resources would better enable them to initiate discussions about anal pleasure and health. The training included a participant manual but no client-facing materials, like brochures or a website. Participants requested these materials as vehicles to foreshadow for clients during clinical encounters the relevance of discussions and to market their services in online forums. However, in recent years, the Chinese regulatory environment has tightened, with anti-pornography laws and public morality regulations being wielded to censor or shut down online platforms with sexual health content and police raids of organizations providing health education and confiscation of their printed materials [48, 49]. This makes the availability of credible client-facing materials particularly important, but also more challenging to deliver.

The training itself also lacked specific guidance and practice (e.g., how to start a discussion; how to draw out shy clients; how to lessen the likelihood of perceptions of sexual harassment). The materials provided by the training were, in some cases, insufficient to support accurate, sex-positive discussions about anal sex, as evidenced by a few participants whose descriptions of their intended approaches to integrate knowledge into practice strayed far from the recommended practices of the training. To further enable health workers, changing antecedents (e.g., the creation of client-facing materials to serve as antecedent visual and virtual objects to prompt discussion) and providing practical social support (e.g., the provision of exemplar language for providers struggling to broach discussion and provide consistent messaging) are warranted. Shifting stigma requires multilevel approaches [50] and restructuring aspects of a health workers’ environment that do not yet sufficiently encourage discussion (e.g., intake forms, organizational context and resources) may further enable proactive discussion, though the current context in China may make this challenging to implement. Additionally, our findings suggest the importance of social reinforcement, but the intervention itself focused on single-agent providers, albeit some who worked at higher levels of organizational and public health influence. Future revisions to the intervention could leverage the relative power of individuals to enact changes within their organizational systems to shift practice within and across levels of influence [50].

Our findings should be considered in light of several limitations. The preliminary impact within our pilot study may have been powered by a highly stigmatizing social context in China, where access to information about same-sex behavior is restricted. This social context of low knowledge about anal pleasure and health may have inflated the effects of the intervention, for example toward improved knowledge, such that we cannot generalize about preliminary impact across country settings where information may be more readily available. However, across the globe anal sex remains highly stigmatized [1, 13, 51, 52], suggesting both the need and potential impact in other contexts where access to accurate, reliable information about anal sex remains limited or restricted. Our study found statistically significant changes after the intervention; however, participants’ initial (baseline) ratings were already moderate to high. These high baseline ratings may have been overestimates—possibly due to the Dunning-Kruger effect [37], where individuals overrate their abilities because they lack full awareness of their deficits. The intervention may have made participants more aware of their actual skill levels. The exclusive reliance on self-report measures makes the findings susceptible to this effect. For example, self-report assesses perceived skill rather than actual skill, and participants often cannot accurately assess their deficits until an intervention provides them with a proper framework for what skillful communication looks like. To better assess changes in future studies, we could compare our current assessment approach to assessment with a retrospective pre-test, where participants rate themselves after the intervention, once they have a clearer understanding of the subject matter. A complementary approach would be to triangulate findings by introducing objective assessments. An additional limitation is the likelihood of social desirability bias. Our participants knew that the evaluation was being conducted by members of the same team that sponsored the intervention. They may have then been susceptible to over-reporting the positive impacts of the training. Still, participants shared deficits as well, an indication that at least some responded candidly despite the risk of social desirability bias. Our pilot study was not powered for inferential statistics; statistical effects, both significant and insignificant, may be attributable to chance and to confounds not included in the analyses. Nonetheless, qualitative findings about the impact of the training suggest that the workshop meaningfully and positively influenced mechanisms of action in the pathway toward discussion about anal pleasure and health.

## Conclusion

Understanding mechanisms can inform further refinement of an intervention to increase theoretical rigor and, ideally, impact [53]. To this point, the intervention has since been refined by one of the first authors (BAK), based on the findings of this and other preliminary studies. While our study in China detected positive outcomes, future studies should seek to evaluate the intervention across contexts and with more robust study designs to assess causality. Indeed, a more holistic intervention, adding environmental restructuring through the provision of client-facing materials and quality improvement meetings, is being piloted in the US to assess preliminary effectiveness and activation of mechanisms of action (NCT04779736). Mitigating Sexual Stigma Within Healthcare Interactions Improve Engagement of MSM in HIV Prevention. ClinicalTrials.gov Identifier: NCT04779736. Available at: https://clinicaltrials.gov/study/NCT04779736. Our hope is that our pilot study contributes to the advancement of stigma research, by demonstrating how to assess and evaluate mechanisms of action early in the development of an intervention.

## Footnotes

1 At the time this research was conducted, the term “MSM” was commonly used in China to refer to gay and bisexual and other men who have sex with men. Therefore we maintain the use of this term when reporting on scale items and when including direct quotes. However, in the manuscript itself we use the term gay and bisexual men.
